# Evaluation of nutrition literacy and nutrition knowledge level in nursing students: a study from Turkey

**DOI:** 10.1186/s12912-022-01146-z

**Published:** 2022-12-16

**Authors:** Özge Mengi Çelik, Remziye Semerci

**Affiliations:** 1 Faculty of Health Sciences, Department of Nutrition and Dietetics, Trakya University, Edirne, Turkey; 2 School of Nursing, Koç University, Istanbul, Turkey

**Keywords:** Nutrition knowledge, Nutrition literacy, Nursing student

## Abstract

**Objective:**

The determination of nutritional knowledge and nutrition literacy among nursing students will enable nursing departments to establish the needs and solutions to enhance nutrition education in their education programs. Therefore, this study is aimed to evaluate the nutrition literacy and nutrition knowledge level of nursing students.

**Method:**

The study data were collected with ‘Information Form’, ‘Anthropometric Measurements’, ‘Nutrition Knowledge Level Scale for Adults’, and ‘Evaluation Instrument of Nutrition Literacy on Adults’. Analyzes were performed using descriptive and nonparametric tests.

**Results:**

The score of nutrition knowledge is 56.6 ± 6.8 and 50.5% of them have a good nutrition knowledge level. The total nutrition literacy score is 28.6 ± 4.4 and 91.6% of them have a sufficient nutrition literacy level. It was no significant difference between students’ characteristic features and nutrition knowledge score and nutrition literacy total score (*p* > 0.05). There was a statistically significant positive correlation between the nutrition knowledge score and the nutrition literacy total score and the nutrition literacy sub-sections scores (*p* < 0.05).

**Conclusion:**

It has been determined that the nutrition knowledge and nutrition literacy levels of nursing students correlated with each other. To improve students’ nutrition knowledge levels, as well as to improve their nutrition literacy and prevent non-communicable diseases nutrition lessons should be included in the curriculum.

## What is known about the topic?

- ▪ Diet-related diseases as non-communicable diseases are highest proportion of overall mortality.
- ▪ Nutrition literacy and nutrition knowledge decrease risk of the diet-related diseases.
- ▪ Nurses and nursing students have a more important role to increase the public’s level of nutrition literacy and nutrition knowledge.

## What does this paper add?

- ▪ It is little known about the relationship between nursing students’ nutrition literacy and nutrition knowledge
- ▪ The nutrition knowledge and nutrition literacy levels of nursing students correlated with each other.
- ▪ Developing students' nutritional knowledge levels and nutritional literacy will contribute to the prevention of non-communicable diseases.

## Introduction

Poor nutrition quality and unhealthy eating habits are major risk factors for chronic illnesses such as obesity, diabetes, cardiovascular disease, and several malignancies [1]. Therefore, dietary risks have received more attention during the last decade [2]. Dietary risk factors were responsible for 7.9 million deaths and 187.7 million disability-adjusted life years in 2019 [2]. Additionally, non-communicable diseases (NCDs) accounted for the highest proportion of overall mortality (74.4%) in 2019, growing by 20.5 percent from 2009 to 2019, meaning 7.1 million more deaths in 2019 compared to 2009 [3]. The World Health Organization has determined many policies regarding NCDs, but in a study covering 194 countries, it was reported that one-third of the 19 policies proposed in 2020 (32.8%) were implemented [4]. Therefore, it is critical to promote approaches that take into account both the environment and the consumption of healthy foods to prevent NCDs and the morbidities caused by these diseases [5]. One of the most important strategies is to increase the nutrition literacy and nutritional knowledge of society.

Nutrition literacy and nutrition knowledge have emerged as critical components in promoting and maintaining healthy eating behaviors [6, 7]. Health literacy is defined as the degree to which individuals obtain, understand, and use basic health information and services to make informed health decisions [8, 9]. Nutrition literacy is a special component of health literacy [7]. Even though nutrition literacy has developed as a unique type of health literacy, academics continue to focus on component skills and capacities in light of conversations about what it means to be nutrition and health literate [10]. In general, it is defined as an individual's capacity to obtain, process, and understand basic nutritional information necessary to make appropriate dietary decisions [1, 11]. Individuals with adequate nutrition literacy have basic nutritional knowledge and have the skills to understand information about nutrients and food groups, read the food label and do portion control. It is very important to increase the level of knowledge about nutrition in the prevention and reduction of nutrition-related NCDs [12, 13]. In this context, it is critical that nurses, the health professionals, determine the nutrition literacy and nutrition knowledge level of the society and provide consultancy and training in this direction.

Nurses play an important role in protecting and improving health and increasing the quality of life [14]. It is an important group of healthcare professionals involved in the delivery of nutritional care to patients and they work closely with patient groups and have opportunities to identify at-risk health behaviors. Nurses who provide nutritional care to patients need to determine their nutrition knowledge levels and accordingly, individuals with low nutrition knowledge need to improve their nutrition knowledge [15, 16]. Also, nurses have an increasingly prominent role in NCDs prevention and management. Nurses who play a role in the prevention and treatment of NCDs should have a high level of nutrition literacy and be able to transform this knowledge into their lifestyle practices. However, studies show that nurses are at risk of being overweight and obese [17–19]. It is very important to prevent this situation by increasing the nutrition literacy and nutrition knowledge of nurses beginning with undergraduate education [17, 20].

In Turkey, studies that examine nutrition literacy and nutrition knowledge among nursing students are limited. Considering the nursing students, who are defined as the young population, it is very important to have a high level of nutrition knowledge in this age group for the society of the future to be healthy. In this context, this is aimed to examine the nutrition literacy and nutrition knowledge level of nursing students.

### Study questions

1. What is the nutrition literacy level of the nursing students?
2. What is the nutrition knowledge level of the nursing students?
3. Is there a relationship between nutrition literacy and the nutrition knowledge level of nursing students?

## Method

### Study design

This cross-sectional and descriptive study design was used in this study. The study was conducted with 309 nursing students between January-March 2022 at the Faculty of Health Sciences, Department of Nursing.

### Participants

The sample comprised 690 nursing students from the Faculty of Health Sciences, Department of Nursing at a university in Turkey. The G*Power software (Version 3.1.9.6) was used to analyze the sample's size [21]. Because there is no similar study, the effect size of the study was aimed to be a poor or medium correlation between nursing students’ nutrition literacy and nutrition knowledge level. Based on the effect size |ρ|= 0.20, correlation: point biserial model, according to the with two tail, α err prob = 0.05, Power (1-β err prob) = 0.95, the sample size was calculated 262. Considering the data loss, the sample was increased by 20% and the study was required 314 nursing students. Five students were excluded from the study because they filled in the questionnaires and scales incompletely. Therefore, the study was completed with 309 nursing students. The post-doc analysis was performed after the study, with correlation: point biserial model, according to the with two tail, effect size |ρ|= 0.45, α err prob = 0.05, and the sample = 309, the power of the study was determined to 99%.

The inclusion criteria of the study were being nursing students, being able to speak Turkish, volunteering to participate in the research. The exclusion criteria were filling in the data in completely and wanting to leave the research at any stage of the research.

### Ethical considerations

Ethical approval with the decision number 25/23 dated 27.12.2021 from Trakya University Faculty of Medicine Dean's Office of Ethics Committee for Non-Invasive Scientific Research before data collection. The researchers wrote to the nursing students the aims and methods of this research in an online google survey, explaining that confidentiality was protected and participation in the research was completely voluntary. Informed consent was obtained from all participants included in the study. Individuals who ticked the "I consent to participate in this study voluntarily" tab at the beginning of the web-based questionnaire were included in the study. In addition, the students were informed that they could leave the research at any time without giving any reason. All procedures in the study were carried out following the Declaration of Helsinki.

### Data collection

The study data were collected with ‘Information Form’, ‘Anthropometric Measurements’, ‘Nutrition Knowledge Level Scale for Adults (NKLSA)’ and ‘'Evaluation Instrument of Nutrition Literacy on Adults (EINLA)’. Researchers create data collection tools through google surveys. With the help of the academic staff in the nursing department, necessary explanations were made and shared with the students in the WhatsApp groups of each course. This data collection method has been preferred in order not to exchange materials and maintain social distance in accordance with the pandemic rules.

### Data collection measurements

#### Information form

The form was created by the researchers according to the literature [17, 22, 23]. The socio-demographic characteristics of the individuals were asked about their age, gender, what grade they were studying in, and whether they had taken any courses on nutrition at the university. From the nutritional habits of the individuals, the main meal and snack consumption status of the individuals was evaluated. The form consisted of seven questions.

#### Anthropometric measurements

Anthropometric measurements (body weight and height) of the individuals were questioned based on the statement. Individuals were informed about how to take anthropometric measurements in the questionnaire form. Body mass index (BMI) was calculated by dividing the body weight by the square of the height [24]. If the body mass index is less than 18.50 kg/m^2^, individuals are classified as underweight, between 18.50–24.99 kg/m^2^ as normal (healthy weight), between 25.0–29.99 kg/m^2^ as overweight, and 30.0 kg/m^2^ or higher as obese [25].

#### Nutrition knowledge level scale for adults

The first part of the 'Nutrition Knowledge Level Scale for Adults' was used to evaluate the nutritional knowledge level. The scale was developed by Batmaz and Güneş [26] in 2018 and its Turkish reliability and validity study was conducted. The first part of the scale, 'Basic Nutrition Information', consists of 20 questions. The answers were scored as ‘I strongly agree’ 4 points, ‘agree’ 3 points, ‘undecided’ 2 points, ‘disagree’ 1 point and ‘strongly disagree’ 0 points. Questions 1, 3, 6, 8, 13, 16, 19 and 20 are scored in reverse on the scale. The maximum score that can be obtained under the heading of basic nutrition knowledge is 80. The knowledge level of the participants with a basic nutrition knowledge score of less than 45 is evaluated as ‘bad’, the knowledge level of those between 45–55 points as ‘medium’, those between 56–65 points as ‘good’, and those with 65 points above as ‘very good’ [26].

#### Evaluation instrument of nutrition literacy on adults

The nutrition literacy status of individuals was determined with the 'Evaluation Instrument of Nutrition Literacy on Adults’. The scale was developed by Cesur et al. [12] and its Turkish validity and reliability study was conducted. The scale consists of 35 questions. Each correct answer in the scale is worth ‘1’ and wrongly answered questions are worth ‘0’. The total score of the scale ranges from 0 to 35 points. Nutrition literacy level is classified as ‘insufficient’ between 0–11 points, ‘borderline’ between 12–23 points, and ‘sufficient’ between 24–35 points out of the total score. The scale comprised five sections.

##### First section

There are 10 questions about ‘general nutrition information. Nutrition literacy level is scored between 0–3 points as 'insufficient', between 4–7 points as ‘borderline’ and between 8–10 points as 'sufficient'.

##### Second section

There are 6 questions about ‘reading comprehension and interpretation’. Nutrition literacy level is scored between 0–2 points as 'insufficient', between 3–4 points as ‘borderline’ and between 5–6 points as 'sufficient'.

##### Third section

There are 10 questions about ‘food groups’. Nutrition literacy level is scored between 0–3 points as 'insufficient', between 4–7 points as ‘borderline’ and between 8–10 points as 'sufficient'.

##### Fourth section

There are 3 questions about ‘serving sizes’. Nutrition literacy level is scored 0–1 point as 'insufficient', 2 points as ‘borderline’ and 3 points as 'sufficient'.

##### Fifth section

There are 6 questions about ‘reading food labels and basic mathematics’. Nutrition literacy level is scored between 0–2 points as 'insufficient', between 3–4 points as ‘borderline’ and between 5–6 points as 'sufficient' [12].

### Statistical analysis

The analyses eliminated cases with missing data for the primary research variables. The Statistical Package for the Social Sciences (version 22.0) software was used for analyses. Data were evaluated with descriptive statistics such as mean, standard deviation, number and percentage. Distribution analysis of the data was performed using the histogram, coefficient of variation ratio, Skewness, Kurtosis and Kolmogorov–Smirnov tests. Mann–Whitney U test and Kruskal Wallis test were used in independent groups for comparison. The relationship between numerical variables was evaluated with the Spearman correlation coefficient. A p-value of less than 0.05 was considered to be statistically significant.

## Results

The general characteristics of the nursing students are given in Table 1. A total of 309 (53 male, 256 female) nursing students participated in the study. The average age of the students is 20.2 ± 1.3 years, and the average BMI is 22.4 ± 3.7 kg/m^2^. 69.9% of the students have a healthy body weight. 27.2% of the students participating in the study had taken a course on nutrition at the university. The number of main meals consumed is 2.4 ± 0.5 and the number of snacks is 1.5 ± 1.0 by the students.

**Table 1:** General characteristics of nursing students

**Variables**	${\varvec{M}}{\varvec{e}}{\varvec{a}}{\varvec{n}},\boldsymbol{ }{\varvec{S}}{\varvec{D}}$
**Age (years)**	20.2 ± 1.3
**Number of main meals**	2.4 ± 0.5
**Number of snacks**	1.5 ± 1.0
**Gender (n, %)**	
Female	256 (82.8)
Male	53 (17.6)
**Studying in grade (n, %)**	
1st grade	89 (28.8)
2nd grade	105 (34.0)
3rd grade	73 (23.6)
4th grade	42 (13.6)
**Take a nutrition course (n, %)**	
Yes	84 (27.2)
No	225 (72.8)
**Body mass index (kg/m**^**2**^**) (n, %)**	22.4 ± 3.7
Underweight (< 18.5 kg/m^2^)	40 (12.9)
Healthy weight (18.5–24.99 kg/m^2^)	216 (69.9)
Overweight (≥ 25.00–29.99 kg/m^2^)	40 (12.9)
Obese (≥ 30.0 kg/m^2^)	13 (4.2)

***SD*** Standard deviation

The nutrition knowledge levels of individuals are given in Table 2. The mean score for nutrition knowledge is 56.6 ± 6.8 points. 6.1% of the students have a bad nutrition knowledge level, 34% have a medium nutrition knowledge level, 50.5% have a good nutrition knowledge level and 9.4% have a very good nutrition knowledge level.

**Table 2:** Nutrition knowledge level of nursing students

**Variables**	${\varvec{M}}{\varvec{e}}{\varvec{a}}{\varvec{n}},\boldsymbol{ }{\varvec{S}}{\varvec{D}}$
**Nutrition knowledge score**	56.6 ± 6.8
**Classification of nutrition knowledge level**	**n, %**
Bad (< 45 points)	19 (6.1)
Medium (45–55 points)	105 (34.0)
Good (56–65 points)	156 (50.5)
Very good (> 65 points)	29 (9.4)

***SD*** Standard deviation

The nutrition literacy status of individuals is given in Table 3. The total nutrition literacy score is 28.6 ± 4.4 points and 91.6% of the students have a sufficient nutrition literacy level according to the total score. When the scores obtained according to the sub-sections of the scale were evaluated, it was determined that the majority of the students (73.1%, 82.8% and 92.6%, respectively) had sufficient nutrition literacy levels in the sections of general nutrition information, reading comprehension and interpretation, and food groups. While only 13.6% of the students have sufficient nutrition literacy about serving sizes, 52.8% of the students have sufficient nutrition literacy levels in reading food labels and basic mathematics.

**Table 3:** Evaluation of nutrition literacy status of nursing students

**Variables**	${\varvec{M}}{\varvec{e}}{\varvec{a}}{\varvec{n}},\boldsymbol{ }{\varvec{S}}{\varvec{D}}$
**Nutrition literacy total score**	28.6 ± 4.4
**Classification of nutrition literacy level**	
İnsufficient (0–11 points)	4 (1.3)
Borderline (12–23 points)	22 (7.1)
Sufficient (24–35 points)	283 (91.6)
**General nutrition information score**	8.2 ± 1.6
İnsufficient (0–3 points)	5 (1.6)
Borderline (4–7 points)	78 (25.2)
Sufficient (8–10 points)	226 (73.1)
**Reading comprehension and interpretation score**	5.1 ± 1.0
İnsufficient (0–2 points)	10 (3.2)
Borderline (3–4 points)	43 (13.9)
Sufficient (5–6 points)	256 (82.8)
**Food groups score**	9.1 ± 1.6
İnsufficient (0–3 points)	10 (3.2)
Borderline (4–7 points)	13 (4.2)
Sufficient (8–10 points)	286 (92.6)
**Serving sizes score**	1.6 ± 0.7
İnsufficient (0–1 point)	143 (46.3)
Borderline (2 points)	124 (40.1)
Sufficient (3 points)	42 (13.6)
**Reading food labels and basic mathematics score**	4.5 ± 1.2
İnsufficient (0–2 points)	16 (5.2)
Borderline (3–4 points)	130 (42.1)
Sufficient (5–6 points)	163 (52.8)

***SD*** Standard deviation

The nutrition knowledge level and nutrition literacy status of individuals according to some parameters are given in Table 4. No significant difference was found in the nutrition knowledge score and nutrition literacy total score of the students according to gender, class, taking nutrition course and BMI classification (*p* > 0.05).

**Table 4:** Evaluation of nutrition knowledge level and nutrition literacy status according to some parameters

Variables	Nutrition knowledge level	*p* value	Nutrition literacy	*p* value
**Gender**				
Female	56.4 ± 6.8	0.148^a^	28.8 ± 4.2	0.073^a^
Male	57.7 ± 6.9		27.6 ± 5.2	
**Studying in grade**				
1st grade	57.2 ± 6.5	0.162	28.9 ± 3.9	0.247
2nd grade	56.2 ± 7.5		27.7 ± 5.3	
3rd grade	55.7 ± 6.4		29.3 ± 3.6	
4th grade	58.0 ± 6.1		29.2 ± 3.3	
**Take a nutrition course**				
Yes	57.7 ± 7.1	0.143^a^	29.0 ± 3.8	0.399^a^
No	56.2 ± 6.7		28.5 ± 4.6	
**Body mass index (kg/m**^**2**^**)**				
Underweight	55.9 ± 7.1	0.640	29.2 ± 2.5	0.124
Healthy weight	56.9 ± 6.9		28.3 ± 4.7	
Overweight	55.9 ± 6.2		29.6 ± 4.6	
Obese	56.6 ± 6.5		29.7 ± 2.0	

^a^Mann-Withney U test, otherwise data expressed as Kruskal Wallis test, *Significant at *p* < 0.05

The relationship between nutrition knowledge level and nutrition literacy status is given in Table 5. There was a statistically significant positive correlation between the nutrition knowledge score and the nutrition literacy total score and the nutrition literacy sub-sections.

**Table 5:** Evaluation of the relationship between nutrition knowledge level and nutrition literacy status

Variables	Nutrition knowledge score
Nutrition literacy total score	*r* = 0.451 *p* < 0.001^*^
General nutrition information score	*r* = 0.413 *p* < 0.001^*^
Reading comprehension and interpretation score	*r* = 0.341 *p* < 0.001^*^
Food groups score	*r* = 0.136 *p* = 0.017^*^
Serving sizes score	*r* = 0.184 *p* = 0.001^*^
Reading food labels and basic mathematics score	*r* = 0.284 *p* < 0.001^*^

Data expressed as non-parametric correlation of Spearman-Rho

^*^Significant at *p* < 0.05

In addition, the relationship between BMI and nutritional literacy and nutritional knowledge level was evaluated, and no statistically significant difference was found between BMI and nutritional literacy total score (*p* > 0.05) and nutrition knowledge score (*p* > 0.05). (data not shown in the table).

## Discussion

This study is very important in terms of defining the nutrition literacy and nutrition knowledge levels of nursing students and examining the relationship between nutrition literacy and nutrition knowledge levels. The important finding is it was found a significant correlation between nursing nutrition literacy and nutrition knowledge levels. It was determined that as the nutrition knowledge levels of nursing students increased, the nutrition literacy levels of nursing students also increased.

In recent years, the dramatic increase in diet-related NCDs has been linked to obesogenic settings, which promote the excessive consumption of unhealthy foods while limiting opportunities for physical exercise [2, 4, 5]. For this reason, it is critically important to inform society about nutrition and to implement interventional strategies that will increase the level of nutrition literacy [4, 14]. It is important that nurses, who have an important role in the implementation of strategies to improve public health and in making decisions, are given training that will increase their nutrition knowledge and nutrition literacy level, starting from their undergraduate education [27]. Thus, nursing students with high nutrition knowledge and nutrition literacy will know to consult society as the nurses of the future before they graduate. In this study, it was found that 50.5% of the nursing students had good nutrition knowledge levels. Unfortunately, 40.5% of them had medium or bad nutrition knowledge levels. In a study by Chepulis and Mearns [17], they stated that nursing students’ had bad nutrition knowledge levels. In a systematic review, it was reported that nurses' nutrition knowledge has increased with experience, they have poor knowledge of providing medical therapy [14]. Similarly, Cho et al. [22], examined nurses’ e-health literacy and they found that nurses had poor knowledge about nutrition. According to all the studies’ results, it was said that nurses and nursing students have moderate or poor nutrition knowledge. In Turkey, most of the nursing curriculum has ‘Nutrition Lessons’, but most of them are related to diseases and the lesson’s duration is two or three hours a week. Our study showed that this lessons’ content should be revised, and it is included general nutrition knowledge to support public health not just for unhealthy people also it is covered, healthy people. This highlights nutrition content as being critical for inclusion in a nursing curriculum.

In the present study, most of the nursing students (91.6%) have sufficient nutrition literacy. However, only 13.6% of students have adequate nutrition literacy in portion sizes and 52.8% in food label reading and basic math. Mehri et al. [28], stated that nursing students’ nutrition literacy is low. In a study that examined nurses’ e-health literacy, it was emphasized that while nurses have a high level of e-health literacy, they have low nutrition literacy [22]. Nurses, being the main group of health professionals [29], are well placed to manage and support patients' basic nutrition care requirements in their various work environments and cultures. In hospitals, this may involve eating problems, dehydration, and/or malnutrition [30], which adds to longer hospital stays and an increased risk of death if left untreated [31]. Therefore, improving patients’ health outcomes is an important responsibility of the nurses [27]. However, according to Chao et al. [32], student nurses' knowledge on this subject is quite limited. Insufficient nutrition literacy has been one of the main barriers to providing adequate, high-quality nutritional care, to their patients. It is very important to plan trainings after undergraduate education to increase the confidence and motivation of nursing students to provide nutritional care to patients [32, 33]. Therefore, nursing students’ nutrition literacy should be increased during the undergraduate and post-graduate to increase public health outcomes and life quality of patients.

It was emphasized that nutrition knowledge and nutrition literacy correlated with each other [27]. In this context, in this present study, it was found a positive correlation between the nutrition knowledge score and the nutrition literacy total score and sub-sections scores. Similar to our results, Kim et al. [34], stated that the dietary attitudes of the nursing students’ correlated positively with nutrition knowledge. Liao et al. [35], reported that nutrition literacy explained 17.2% of the total variance of healthy-eating behaviors of college students. Uysal et al. [36], conducted their study with 905 students in nursing, law and Islamic sciences departments and it was determined that health literacy correlated with nutrition knowledge. In a line of the literature, it can be said that, if nurse educators increase nursing students’ nutrition knowledge, students’ nutrition literacy will increase. Current undergraduate nursing nutrition education is inadequate to meet the requirements of nurses as future health professionals in providing nutritional care according to the needs of patients or themselves. It is critical to prepare for the training required to support nurses' and their own long-term health, as well as to develop their professional competence to meet contemporary nutritional concerns [36]. Therefore, nursing students’ ability to obtain, process and understand nutrition information and skills needed to make appropriate nutrition decisions should be improve from the undergraduate and nutrition lesson should be include in the nursing curriculum.

## Conclusion

This research reports the findings of a study that evaluated nursing students' nutrition knowledge levels, as nurses are in a superior position to guide as nutrition instructors and advisors by interacting with their patients in discussions that enhance their knowledge regarding disease prevention and treatment via nutrition. It has been determined that the nutrition knowledge and nutrition literacy levels of nursing students are related to each other, therefore, clinical and general nutrition should be given more place in the curriculum of nurses. The findings of this study show that nursing students need nutrition-related courses in nursing curricula in order to improve their nutritional literacy levels, as well as improve their nutritional knowledge and prevent NCDs. The implications of this study are significant for the nursing curriculum.

## Limitations

This study has some limitations. Firstly, the study included a small sample size. The response rate was also approximately 45% which to an extent may not give a wider representation of the study participants. Secondly, we aimed to evaluate the relationship between nursing students' nutritional knowledge and nutritional literacy. For this reason, we did not add the factors affecting the nutritional knowledge and nutritional literacy levels of nursing students to the questionnaire. It is recommended that future studies examine the factors affecting the nutritional knowledge and nutritional literacy levels of nursing students.

## Data Availability

The datasets generated and/or analysed during the current study are not publicly available due to privacy or ethical restrictions, but are available from the corresponding author on reasonable request.
